# Characterization of Cementation Factor of Unconsolidated Granular Materials Through Time Domain Reflectometry with Variable Saturated Conditions

**DOI:** 10.3390/ma12081340

**Published:** 2019-04-24

**Authors:** Yong-Hoon Byun, Won-Taek Hong, Hyung-Koo Yoon

**Affiliations:** 1 School of Agricultural Civil & Bio-Industrial Engineering, Kyungpook National University, 80 Daehak-ro, Buk-gu, Daegu 41566, Korea; yhbyun@knu.ac.kr; 2 Department of Civil and Environmental Engineering, University of Illinois at Urbana-Champaign, 205, North Mathews Avenue, Urbana, IL 61801-2352, USA; wthong@illinois.edu; 3 Department of Construction and Disaster Prevention Engineering, Daejeon University, Daejeon 300-716, Korea

**Keywords:** cementation factor, dielectric constant, electrical resistivity, saturation, time domain reflectometry

## Abstract

The cementation factor is necessary to determine porosity via the Archie equation, and its range of values has been suggested in many previous studies. However, the cementation factors in the literature are limited to fully saturated conditions, and it may thus be inaccurate to use the same value in other saturation conditions. The objective of this study is to characterize how the cementation factor varies depending on the saturation percentage. In this study, glass beads and soil are selected as the specimens, and two relative density values, 40% and 80%, are selected. Time domain reflectometry (TDR) is used to obtain both the saturation and electrical resistivity of the specimens. TDR is installed in the cell, and fluid is continuously circulated from the bottom to the top of the porous material for 30 min. The estimated saturation increases with time and the electrical resistivity is reduced during the circulation. Finally, the cementation factor at every saturated stage is determined, and the error ratio based on the porosity is calculated to show the importance of the cementation factor. The results show that there is a high error ratio when an unsuitable cementation factor that does not consider the saturation condition is used. This study demonstrates that the method for determining the actual cementation factor using TDR and the Archie equation can be applied in various saturation conditions.

## 1. Introduction

Pore structure plays an important role when considering the characteristics of porous material because it is affected by various physical properties, including density, strength, friction, and water flow [1,2]. Many researchers have tried to study pore structure with experimental techniques that consider component analysis, pore size distribution and the saturated condition. Meanwhile, the geophysical method is an alternative method to determine the contact mechanism in porous materials at a small strain range [3,4].

Electrical resistivity measurement has been a widely used technique because it is an efficient tool with a simple experimental method and high resolution [5]. Electrical resistivity can be obtained through the relationship between the artificially applied current and the voltage formed by the medium properties. The current flows in both the particles and the void space of the porous material, and if the particles are made of a nonconductive material, the current flow depends only on the void characteristics. Therefore, electrical resistivity is closely related to the porosity in a porous material. There have been many studies to determine the porosity in rock [6,7], sand [8,9], clay [10,11] and mixture [5,12]. To convert electrical resistivity into a porosity, two important constants, the tortuosity factor and cementation factor, are required. The tortuosity factor is related to the tortuosity, which is a property of a curve and is defined as the ratio between the mean path length and the straight-line path of a porous material [13]. The tortuosity factor is generally assumed to be a unity [14]. The grain shape, type of grain, pore structure, specific surface area and compaction are parameters that affect the degree of consolidation called the cementation factor [15]. The range of values of the cementation factor for each porous material has been suggested in the literature; however, these values are based on the fully saturated condition, as shown in Table 1. There have been no studies to understand the behavior of the cementation factor with different saturation conditions; therefore, this study is performed to investigate the effect of the saturation condition on the cementation factor.

Time Domain Reflectometry (TDR) was initially used to measure the volumetric water content in porous material with a specifically calibrated equation [16]. To improve the usability of TDR, [17] demonstrated the resolution of TDR in a spatially distributed area, and [18] suggested a dual reflection analysis for providing the embedded information of the signal. The electromagnetic wave is attenuated when it encounters other areas of impedance [19]. Consequently, the technique was extensively used to understand soil behaviors, including for the measurement of the scour depth [20], internal structure change in an embankment [21], and detection of necking in a bored pile [22]. Therefore, in this study, TDR was also selected to obtain the volumetric water content and electromagnetic wave attenuation for estimating the cementation factor.

The theoretical background for obtaining porosity from electrical resistivity is introduced, and the method for predicting the cementation factor with the consideration of saturation using time domain reflectometry (TDR) is explained. The laboratory test for measuring electrical resistivity and saturation is also described. Finally, the behavior of the cementation factor under various saturation conditions is discussed, and the error ratio based on the porosity is also demonstrated.

## 2. Background Theory

Electrical resistivity, which is an inherent property of a material, is the physical quantity measuring the impedance to the current flow, and it is also the reciprocal of electrical conductivity. It is mathematically composed of functions involving the particles and electrolyte of the porous material and the specific surface area [23]:

(1) $$\frac{1}{\rho_{PM}}=(1-n)\cdot \frac{1}{\rho_{P}}+n\cdot \frac{1}{\rho_{EL}}+(1-n)\cdot \frac{\gamma_{P}}{g}\cdot \lambda_{\mathrm{ddl}}\cdot S_{a}$$

where ρ_PM_ (Ω·m) denotes the electrical resistivity of porous material, and ρ_P_ (Ω·m) and ρ_EL_ (Ω·m) denote the electrical resistivities of the particle and electrolyte, respectively. n (-), γ_P_ (N/m^3^), g (m/s^2^), λ_ddl_ (S) and S_a_ (m^2^/kg) indicate the porosity, unit weight, coefficient of gravity, surface conduction and specific surface area, respectively. In general, Equation (1) can be abbreviated as Equation (2), with the neglecting electrical resistivity of the particle in the first term because the soil particle is an electrically non-conductive material; Equation (2) is generally known as the Archie equation [6]:

(2) $$\rho_{\mathrm{PM}}=\alpha \cdot \rho_{\mathrm{EL}}\cdot n^{-\beta }$$

where α and β are the tortuosity factor and cementation factor, respectively. α is taken as the unity; however, β ranges from 1.3–5.12 depending on the material, as shown in Table 1 [10,11].

The fluid in the porous structure has a close relationship to the current flow, and the saturation (S) can be written in terms of the electrical resistivity of the porous material (ρ_PM_), the electrical resistivity at the fully saturated condition (ρ_FS_) and the saturation factor (δ), as shown in Equation (3):

(3) $$S={\left(\frac{\rho_{\mathrm{FS}}}{\rho_{\mathrm{PM}}}\right)}^{\frac{1}{\delta }}$$

where δ denotes the saturation factor. Even though the saturation factor is affected by various conditions, including the material type and wettability, the values are suggested in the range of 2 to 8 in saturated conditions [35]. 

**Figure 1:**
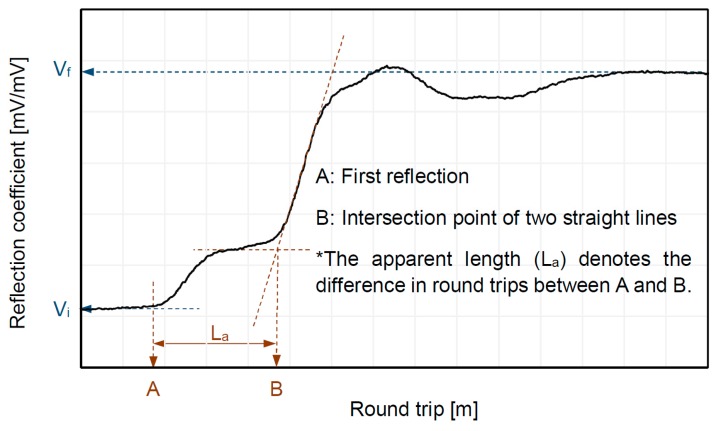
Example of a reflected waveform from TDR.

In this study, time domain reflectometry (TDR) is used to obtain the electrical resistivity and saturation of the porous material. TDR measures the reflected signal of an electromagnetic wave, which is attenuated by the conductive medium, and the degree of attenuation in the reflected waveform is related to the electrical resistivity of the medium [17]. Equation (4) is the mathematical relationship through which one obtains the electrical resistivity from TDR [36]. Although there are various ways of obtaining the electrical conductivity with the dielectric constant through TDR, this study selected Equation (4), as it can be easily used, without complicated considerations:

(4) $$\sigma_{PM}=\frac{1}{\rho_{PM}}=\frac{\sqrt{\epsilon_{PM}}}{120\cdot \pi \cdot L}ln\left(\frac{V_{i}}{V_{f}-V_{i}}\right)$$

where L denotes the length of the TDR electrode, and V_i_ and V_f_ are reflection points at the beginning and end of the probe, respectively. ε_PM_ is the dielectric constant of the porous material, which can be calculated using the apparent length (La) and probe length (L) (ε_PM_ = La^2^/L^2^), as shown in Figure 1. Note that the electrical resistivity (ρ_PM_) can be obtained with the reflected waveform from TDR, and that both the saturation factor (δ) and the cementation factor (β) can be estimated from ρ_PM_.

## 3. Specimens

**Figure 2:**
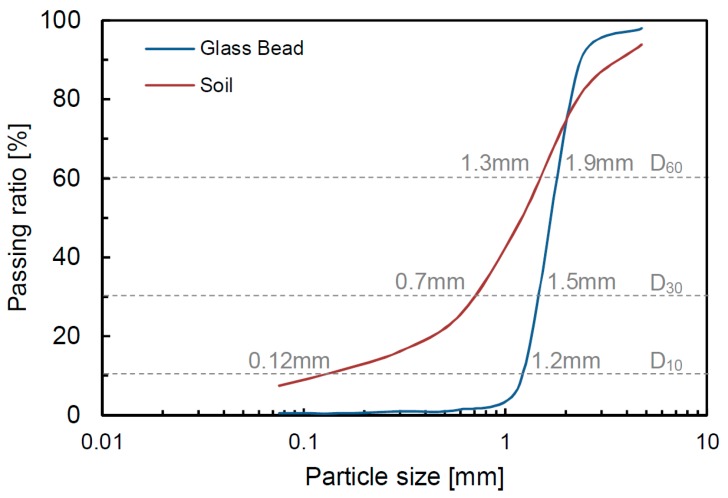
Particle size distributions.

Glass beads and disturbed soil extracted in the field were selected as specimens. Sieve tests were performed, and the particle distribution curves are shown in Figure 2. Figure 2 shows that the glass beads and soil have uniform and well-graded grain size distributions. The diameters corresponding to 10%, 30% and 60% passing ratios are determined to be 1.2 mm, 1.5 mm and 1.9 mm for the glass beads, and 0.12 mm, 0.7 mm and 1.3 mm for the sand, respectively. The uniformity coefficients of the glass beads and soil are calculated as 1.6 and 10.8, respectively. Additionally, the coefficient of the curvature is determined to be 1.0 and 3.1 for the glass beads and soil, respectively. The material properties of the glass beads and soil are also summarized in Table 2.

## 4. Experimental Section

**Figure 3:**
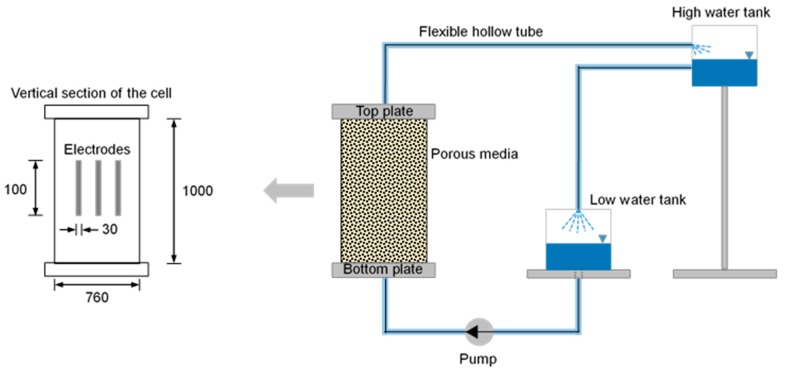
Test configuration for measuring the dielectric constant with various saturation conditions. The water is continuously circulated when the dielectric constant shows the fully saturated value. The dimension unit is mm.

The cell, which is a cylinder, has an inner diameter of 760 mm and a height of 1000 mm. The three electrodes for generating the electromagnetic wave and for receiving the reflected waves were installed into the inner part of the cell, as shown in Figure 3. The inner part of the coaxial cable is connected with the inner single electrode, and the outer part of the coaxial cable is soldered with two left electrodes. The dimensions of the electrode are: 30 mm width, 100 mm height and 1 mm thickness. The TDR electrode was installed at a depth of 500 mm with respect to the center of the electrode. To transmit and measure the electromagnetic wave, the TDR instrument (HL1101, HYPERLABS, Beaverton, OR, USA), which can generate a voltage of 250 mV with a frequency of 10 MHz, was selected. The voltage propagated and reflected through parallel rod electrodes connected with a coaxial cable of 50 Ω. It measures the dielectric constant, which can be converted into electrical resistivity and saturation through Equations (4) and (5), respectively. The fluid is injected from the bottom plate with a pump, and the fluid flows out from the top plate. To improve the electromagnetic wave propagation ability, 0.5 M of a salty solution was used as the fluid. The reflected waveforms were measured every 2 min for 10 min because the measured waveforms were all the same after 10 min. Therefore, the 1st, 2nd, 3rd, 4th, 5th and 6th steps correspond to ≈0, 2, 4, 6, 8 and 10 min, respectively. One can observe that after 10 min the porous media is fully saturated.

**Figure 4:**
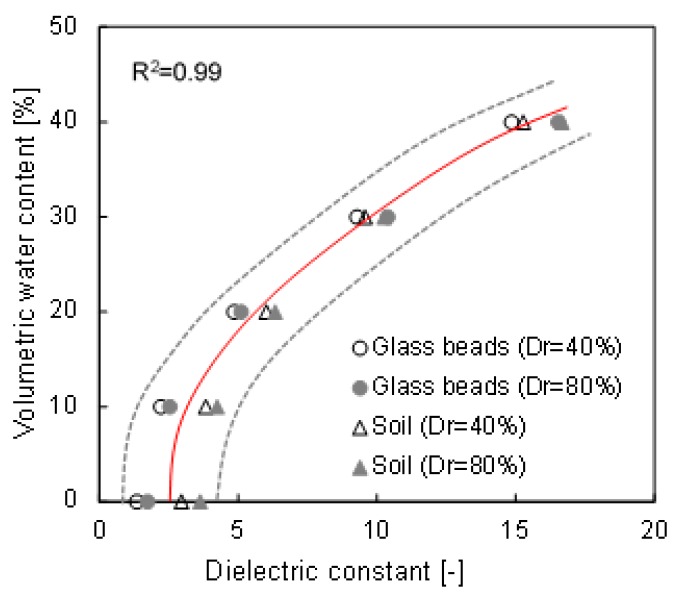
Calibration results between the volumetric water content and the dielectric constant. The gray dotted lines denote the boundary curves, and the red solid line is the averaged curve. The Dr denotes the relative density.

The dielectric constant of water (ε ≈ 80) is higher than that of saturated soil (ε ≈ 2.5–15) [20,37]. Note that the large difference in dielectric constants between the two mediums suggests the possibility of predicting the degree of saturation through measured waveforms from TDR. A pre-experiment was performed to obtain the calibration equation, and the result is presented in Figure 4. The figure shows that the relationship between the dielectric constant and the volumetric water content of each specimen has a similar trend; therefore, the averaged relationship is applied in this study. Equation (5) shows that the measured dielectric constant can be converted into a volumetric water content (θ) using a cubic polynomial regression model, with a root mean squared error (RMSE) of 5.17, 4.28, 4.56 and 4.74 for glass beads Dr = 40%, glass beads Dr = 80%, Soil Dr = 40% and Soil Dr = 80%, respectively. Additionally, the saturation is also obtained through the volumetric water content and porosity (S = θ/n). 

(5) $$\theta =-1.3\cdot 10^{-2}+2.95\cdot 10^{-2}\cdot \varepsilon_{PM}-3.1\cdot 10^{-4}\cdot \varepsilon_{PM}{}^{2}+5.3\cdot 10^{-6}\cdot \varepsilon_{PM}{}^{3}$$

Note that the calibration was performed up to a 40% volumetric water content, because it was difficult to prepare the specimen when the volumetric water content was over 40%.

## 5. Results

**Figure 5:**
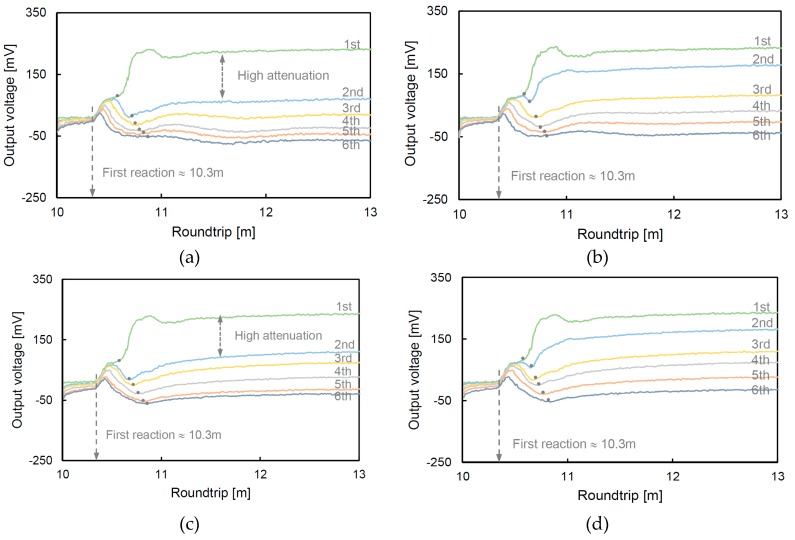
Measured waveforms from TDR: (**a**) glass beads (Dr = 40%); (**b**) glass beads (Dr = 80%); (**c**) soil (Dr = 40%); and (**d**) soil (Dr = 80%). The Dr denotes the relative density, and the dotted points show the reflected positions from the medium.

The measured waveforms for the glass beads and soil are shown in Figure 5. The first reaction of all of the waveforms occurred at similar positions, approximately at 10.3 m, because the characterization of the TDR probe and the length of the specimen connected on the probe were the same. However, the second reflection points, which are shown as dots in Figure 5, vary according to the specimen type and relative density. The round trips of the dotted points gradually increase with time (saturation) due to the attenuation of the electromagnetic wave from ions in the fluid. In particular, the waveforms measured at the 1st and 2nd steps at a relative density of 40% exhibit a higher attenuation than those measured at a high relative density because the low relative density has a high potential to absorb water with high porosity [38].

**Figure 6:**
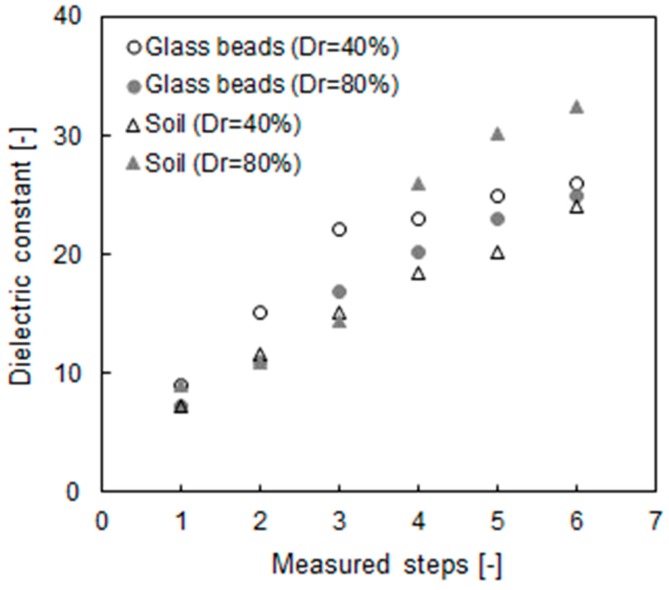
Recorded dielectric constant from TDR with measured steps.

The distribution of the dielectric constants is shown in Figure 6. The initial values of every specimen are ≈9, and the values increased to within 24–32 after an elapsed time. The reason for the increasing dielectric constant is the reduced electromagnetic wave velocity resulting from the adsorption of water [39]. The dielectric constant nearly converged to a similar value according to every specimen after the 5th step, and the result indirectly indicated that the specimen was in a fully saturated condition. Although the measured values of the dielectric constant plotted in Figure 6 are greater than the calibration values shown in Figure 4, Equation (5) is still valid according to previous studies [39,40].

**Figure 7:**
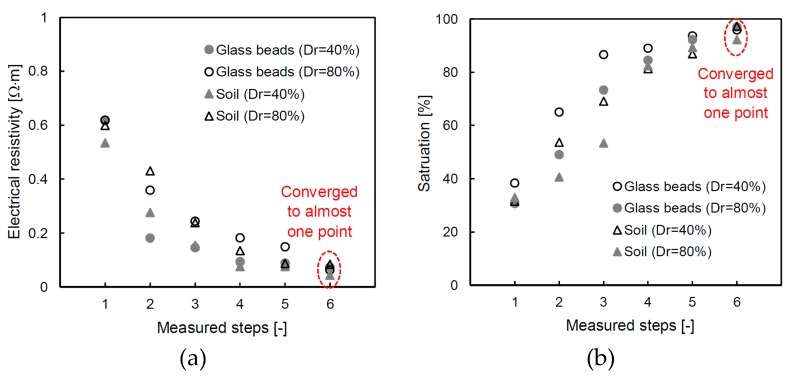
Converted results through the dielectric constant: (**a**) electrical resistivity; and (**b**) saturation.

The changes in the electrical resistivity and saturation calculated in Equations (4) and (5) are plotted in Figure 7; one can see that the electrical resistivity decreased from 0.55 Ω⋅m to 0.055 Ω⋅m, on average, with each step. We consider that the high electrical resistivity appeared due to the content of air in the unsaturated condition. The electrical resistivity of the final stage converges on almost a single value because the electrical resistivity of the fluid dominates the total electrical characterization of the medium as the specimen approaches a 100% saturation [23]. Note that the concentration of the solution is 0.5 M of the salty solution. The saturations of the specimen are estimated to be in the range of 33% to 38% at the initial step, before the values increased to 98% in the final step. The glass bead with a relative density of 40% shows a higher saturated value than that with a relative density of 80% at every step. The result demonstrates that, as the relative density is affected by the saturation speed, the tortuosity, which is the path of the water flow, decreases with an increasing relative density [41]. However, small dielectric constants were observed with the soil that had a relative density of 40% at the 4th, 5th, 6th and 7th steps. The reason for the different behavior is attributed to the content of fine particles, and Ponizovsky et al. [42] showed that it is difficult to determine the calibration constant of TDR when the fine contents increased. In the near future, a study needs to analyze the TDR waveform according to the amount of fine particles. Although the individual behavior is slightly different, in Figure 6 one can see that the overall behavior is consistent. Every saturation at the final step also exhibits similar values and indicates that the circulation time is adequate for achieving a fully saturated condition for every specimen.

## 6. Discussion

### 6.1. Saturation Factor 

**Figure 8:**
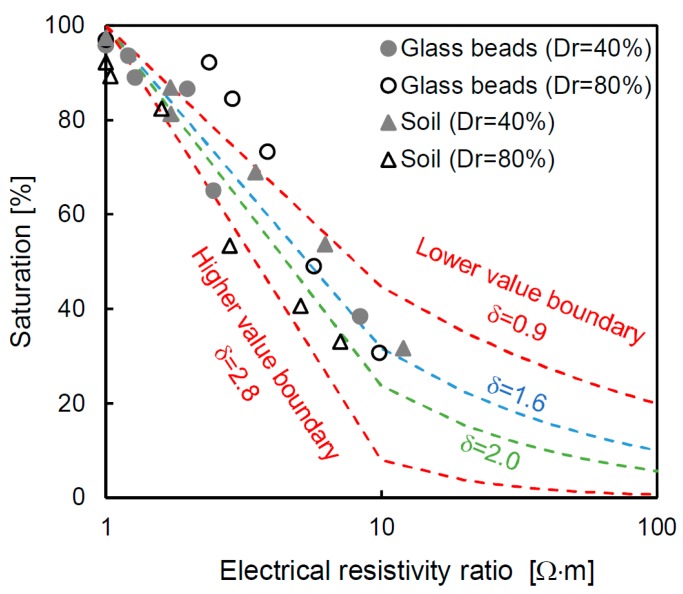
Electrical resistivity ratio versus saturation. The electrical resistivity ratio denotes the relationship between the electrical resistivity of the specimen and the electrical resistivity of the same specimen under the 100% saturated condition. The saturation factors (δ) of 1.6 [43] and 2.0 [35]. The values of the upper and lower boundaries are based on the measured data in this study.

**Figure 9:**
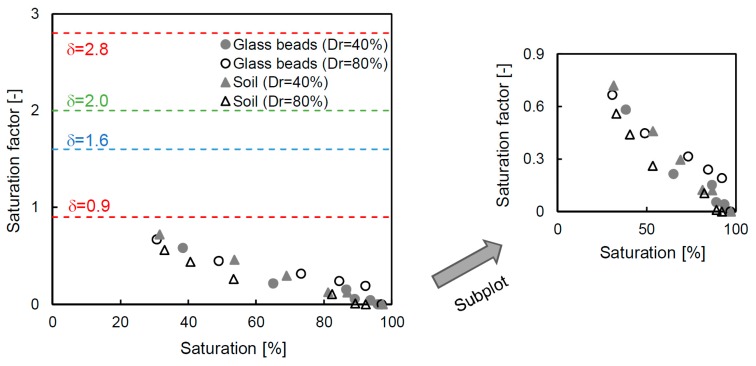
Saturation factors at every saturation step.

The relationship between the saturation and the electrical resistivity ratio is related to the saturation factor, as shown in Equation (3); the values, which are derived from the measured values, are presented on a semi-log scale in Figure 8. The relationship is highly variable, and thus the values are relative to the upper (δ = 2.8) and lower (δ = 0.9) boundaries from the trend line of the measured values. The saturation factors from previous studies of 1.6 [43] and 2.0 [35] are also plotted in this figure, and these values are within the range proposed in this study. The saturation factor exhibits a nonlinear behavior, and there are two different slopes according to the electrical resistivity ratio. Therefore, there is a limit to uniformly using the values derived from one sample in the entire range. Even though [44] suggested an additional constant factor, which they obtained through linear regression results, the method has a limited reliability depending on the degree of saturation of each sample because the method also uses the entire trend line. Thus, the saturation factor is calculated at every stage, considering the specific saturation and electrical resistivity ratio. The detailed saturation factor, which is calculated by inverting Equation (3), is shown in Figure 9. The saturation factor is different for each degree of saturation because the electrical resistivity ratio varies, and therefore deriving a saturation factor using the entire trend of the data includes an error. For the glass beads and soil, the newly calculated saturation factor is in the range of 0.67–0.72 at the initial step, and the value gradually decreases to ≈0 at the fully saturated condition of every specimen. The reason why the saturation factor is almost 0 is because the electrical resistivity ratio is at unity at the 100% saturated condition. The saturation factor in Figure 9 is different from the value derived from the trend line based on δ = 0.9, 1.6, 2.0 and 2.8 (in Figure 8). This tendency suggests that an appropriate saturation factor for each saturation should be determined rather than applying a single value that simply reflects the trend line.

### 6.2. Cementation Factor 

The electrical resistivity of the porous material (ρ_PM_) in Equations (2) and (3) has the same value, and the relationship can therefore be defined in terms of the cementation factor (β), as shown in Equation (6):

(6) $$\beta =\frac{\ln(\rho_{EL})-\ln(\rho_{FS})-\frac{\ln(S)}{\delta }}{\ln(n)}$$

where the parameters are defined as being the same as in Equations (2) and (3).

**Figure 10:**
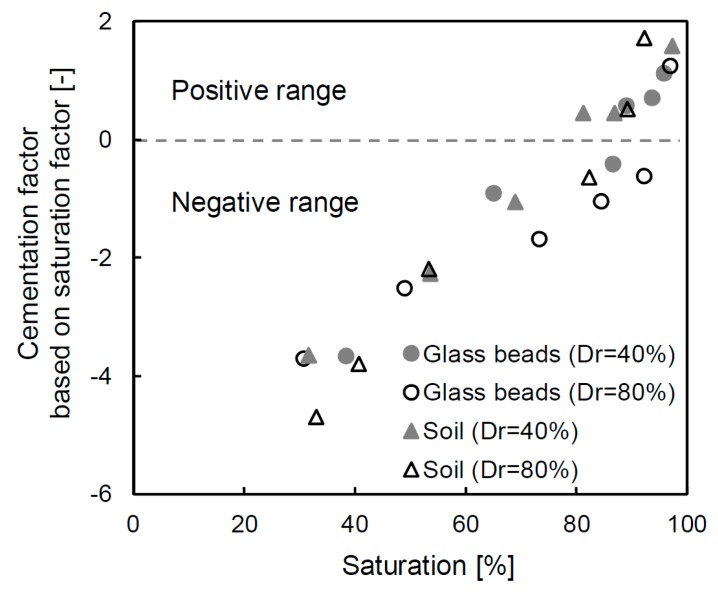
Distribution of the cementation factors based on every saturation factor.

Note that Equation (6) shows that the cementation factor can be predicted when the saturation of the porous material is changed. The calculated cementation factor is shown in Figure 10 and the range of values includes both negative and positive numbers. The detailed cementation factor alternation is also summarized in Table 3. At the initial saturation (≈30–39%), the cementation factor is −3.93 on average, but this changes to a positive value at approximately 90–100% saturation. Even though the degree of saturation exceeds 90%, the cementation factors of glass beads with a 40% and 80% relative density still have negative values. Note that the degree of saturation is a crucial parameter to obtain an accurate cementation factor, and thus the specimen should not assume a fully saturated condition even though it contains water. In particular, the cementation factors of the final saturated condition are 1.12, 1.25, 1.58 and 1.71 for the glass beads of 40% relative density, the glass beads of 80% relative density, the soil of 40% relative density and the soil of 80% relative density, respectively. The estimated cementation factors are similar to those proposed in previous studies [28,34].

The cementation factor shows a higher value when particles are tightly connected to each other [45]. [15] also verified that the cementation factor is affected by compaction, which increases when grains are flattened because the ratio of the throat radius and pore radius is smaller. In this study, the same tendency was observed, and the value of the cementation factor increased by approximately 10% for the glass beads and by 7% for the soil as the relative density increased. Additionally, the cementation factors of the glass beads are smaller than those of the soil. The cementation factor is also related to the pore type because the radius ratio decreases when the specimen approaches a spherical shape due to the small specific surface area. 

### 6.3. Error Ratio of Porosity 

**Figure 11:**
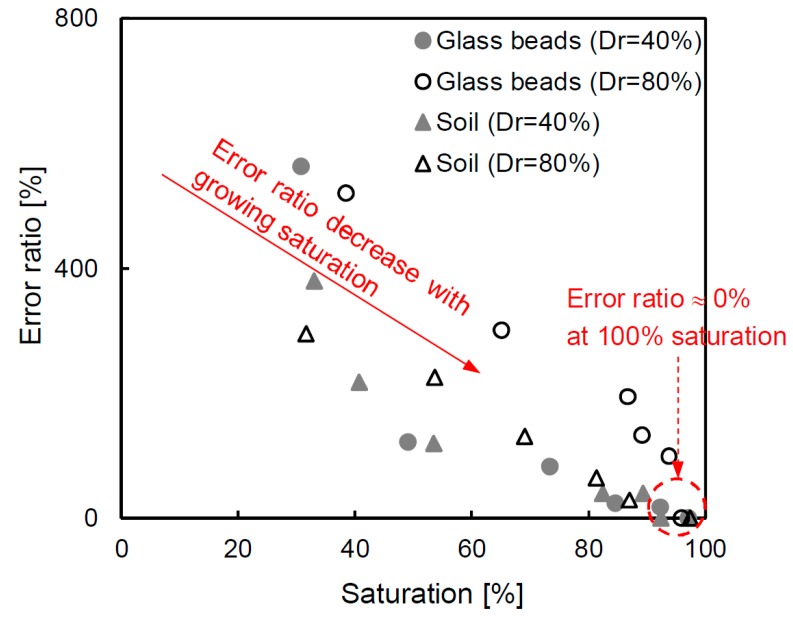
The error ratio based on the porosity when using the cementation factor, deduced by the Archie equation, and assuming a fully saturated condition.

The Archie equation is mainly used to evaluate the condition of the porous medium, and it is also used to calculate the porosity as a design parameter [5,10,11]. However, most studies derived the porosity assuming a 100% saturation without considering the exact saturation value of the sample. If the sample is below the groundwater, it is acceptable to assume a 100% saturation. However, if the sample is at a location close to the groundwater level, it requires an accurate saturation value. Therefore, in this study, the error ratio is estimated through the cementation factor deduced for every specimen at the condition of ≈100% saturation. The error ratio based on the porosity is calculated as shown in Equation (7), and the result is presented in Figure 11.

(7) $$\mathrm{Error}\;\mathrm{ratio}=\frac{n_{\mathrm{inference}}-n_{\mathrm{true}}}{n_{\mathrm{true}}}$$

where n_true_ denotes the porosity calculated from the cementation factor that is estimated using the actual saturation. In addition, n_inference_ indicates the inferenced porosity from the cementation factor based on the 100% saturated condition. The result shows that there is a high error ratio (over 400%) at low saturation conditions, but the error ratio becomes small when the saturation is close to 100%. In particular, in the case of an approximately 100% saturation, the error ratio approaches zero. The high and low error ratios are natural results because the appropriated cementation factor is not applicable. Note that if the exact cementation factor is not calculated using the actual saturation, there will be a large error in the estimation of the porosity of the medium. Overall, the TDR technique, which is applied in this study, is useful to determine a reliable porosity considering the saturation conditions.

## 7. Conclusions

In this study, the distribution of cementation factor values at every saturation stage was determined for unconsolidated granular materials, and the importance of selecting the appropriate cementation factor was also shown using the error ratio based on the porosity. A detailed summary of this study follows:

- Time domain reflectometry is selected to measure the dielectric constant, and the value is converted into the saturation and electrical resistivity of each specimen. Additionally, the saturation factor at every saturation stage is determined from the relationship between the saturation and electrical resistivity ratio.
- The cementation factor, suitable for each degree of saturation, is derived, and the value ranges from negative to positive.
- The error ratio based on the porosity is computed, and a high error ratio indicates an inaccurate saturation range. The results show that the determination of the true cementation factor is necessary for enhancing the reliability.
- Finally, the result shows that the Archie equation can be applied under dried, unsaturated and saturated conditions when the suitable cementation factor is applied.

## Figures and Tables

**Table 1:** Suggested ranges of the cementation factor (β), derived from previous studies.

β	Material Type	Reference
1.6	Natural sand	[6]
2.15	Rock	[24]
1.3	Glass sphere	[25]
1.64–2.23	Natural sediment	[26]
5.12	Sand stone	[27]
1.8-3.0	Kaolinite and sodium montmorillonite	[28]
2.0	Rock with small fissures	[29]
1.3-2.15	Sand	[30]
1.5–2.0	Sea-floor sediment	[31]
1.4–1.66	Quartz and dolomite sand	[32]
1.52–1.58	Natural quartz sand	[33]
1.4–1.6	Marine sands	[8]
1.3–3.3	Sand with shale	[34]

**Table 2:** Material properties of the glass bead and the soil.

Specimens	D_10_(mm)	D_30_(mm)	D_60_(mm)	Cc	Cu	e_max_	e_min_	USCS
Glass bead	1.2	1.5	1.9	1.0	1.6	0.68	0.63	-
Soil	0.12	0.7	1.3	3.1	10.8	0.82	0.59	SW

D_10_, D_50_, and D_60_ denote the diameters at passing percentages of 10, 50, and 60%, respectively. Cc and Cu are the coefficients of the curvature and uniformity, respectively. SW means well-graded sand based on the Unified Soil Classification System.

**Table 3:** Summarized cementation factors for each saturation step.

Saturation		Cementation Factor			
Saturation Range (%)	Specific Saturation Value (%)	Glass Bead Dr = 40%	Glass Bead Dr = 80%	Soil Dr = 40%	Soil Dr = 40%
0~29	-	-	-	-	-
30~39	30 31 33 38	- - - −3.66	−3.70 - - -	- −3.68 - -	- - −4.69 -
40~49	40 49	- -	- −2.51	- -	−3.80 -
50~59	53	-	-	−2.26	−2.18
60~69	65 69	- −0.90	- -	- −1.04	- -
70~79	73	⋅	−1.68	⋅	-
80~89	81 84 86 89	- - −0.40 0.57	- −1.04 - -	0.44 - 0.44 -	−0.63 - 0.52 -
90~100	92 93 95 97	- −0.70 1.12 -	−0.61 - - 1.25	- - - 1.58	1.71 - - -
